# Spatial distribution dynamics for *Epimedium brevicornum* Maxim. from 1970 to 2020

**DOI:** 10.1002/ece3.11010

**Published:** 2024-02-21

**Authors:** Yunfeng Li, Yan Wang, Xiaojuan Du, Chunying Zhao, Ping He, Fanyun Meng

**Affiliations:** ^1^ Hebei Province Key Laboratory of Research and Development of Traditional Chinese Medicine Chengde Medical University Chengde Hebei China; ^2^ Beijing Key Laboratory of Traditional Chinese Medicine Protection and Utilization Beijing Normal University Beijing China

**Keywords:** *E. brevicornum*, SDM, spatial dynamics, Venn diagram

## Abstract

At different time scales, a species will experience diverse distribution changes. For *Epimedium brevicornum* Maxim, the phenomenon is obvious, but the understanding of the spatial dynamics of *E. brevicornum* under distinct time scales is poor. In this study, we modeled the potential distribution for *E. brevicornum* for five time scales, 1970–1979, 1980–1989, 1990–1999, 2000–2009, and 2010–2019, with different occurrence data, and the Kuenm package was used to optimize the parameter combination. Then, SDM tools and a Venn diagram were utilized to simulate the changes in highly suitable areas and spatial dynamics, respectively. Comprehensive results show that temperature seasonality (BIO4, 37.54%) has the greatest effect on the distribution of *E. brevicornum*, followed by minimum temperature (TMIN, 21.42%). The areas of distribution for *E. brevicornum* are 35.06 × 10^5^ km^2^, 25.7 × 10^5^ km^2^, 67.64 × 10^5^ km^2^, 27.29 × 10^5^ km^2^, and 9.87× 10^5^ km^2^, which are mainly concentrated in Gansu, Shaanxi, Shanxi, and Henan, respectively. In addition, the largest regions for expansion, stability, and contraction under various time scales are 5.6 × 10^5^ km^2^, 3.54 × 10^5^ km^2^, and 3.47 × 10^5^ km^2^, respectively. These changes indicate that approximately 7.96% of the regions are highly stable, and three critical counties, Wanyuan, Chenggu, and Hechuan, and Xixiang, have become significant areas for migration. Overall, our results indicate that there are different spatial distribution patterns and dynamics for *E. brevicornum* for different time scales. Given these results, this study also proposes comprehensive strategies for the conservation and management of *E. brevicornum*, which will further improve the current resource utilization status.

## Graphical abstract

The kuenm package was used to optimize the MaxEnt modeling. The temperature seasonality and ground‐frost frequency were regarded as the most important variables. This study determined three significant areas including Qingchuan, Xixiang, and Hanyin, for cultivation such.

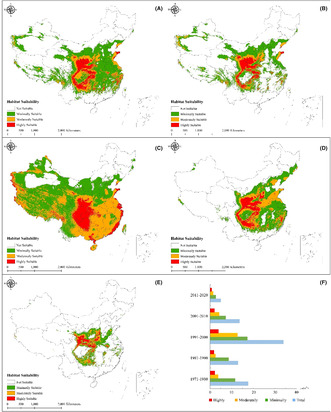

## 1 INTRODUCTION

The distribution dynamics for species under different temporal and spatial scales, resulting from local climate, anthropogenic activities, and interspecific interactions, are a widespread phenomenon (Araújo & Guisan, 2006; Collins & Glenn, 1991; Lortie et al., 2004). To date, addressing these questions in terms of which species will occur after several years remains a changing task because abiotic and biotic environments will influence the distribution of species across different scales (Wisz et al., 2013). The distribution of species at different time scales is determined by two aspects, including their own attributes and external influences. For example, abiotic tolerance, environmental stability, dispersal ability, and biological interactions drive distinct responses for different species to habitat environments (Algar et al., 2009; Briscoe et al., 2021; Guisan & Thuiller, 2005; Napier et al., 2019). Currently, a number of studies focus on discussing the distribution patterns of species under the influence of biotic or abiotic interactions and associating these complex factors with climate change (Pollock et al., 2014; Qiao et al., 2017; Thapa et al., 2018). Sommer et al. (2008) presented spatial–temporal patterns for European late Quaternary *Cervus elaphus* with abundant species occurrence dates and environmental parameters and proposed a conservation strategy. To explore the spatial distribution of three *Coptis* herbs under current and future climate changes, Li et al. (2020) established a distribution pattern using a climate niche model and predicted that future habitat will further contract with global warming. However, these studies have focused on changes in the distribution of species over decades, centuries, and billions of years (Çoban et al., 2020; Zhao, Cui, et al., 2021; Zhao, Deng, et al., 2021), while few studies have followed the spatial dynamics of species at small time scales. Discussions regarding the spatial dynamics of species at large time scales are well reflected in the evolution of species, historical changes, the effects of global warming, and the occurrence of major historical incidents (Elith & Leathwick, 2009; Svenning et al., 2011; Zhang, Liu, et al., 2021; Zhang, Zuo, et al., 2021). In contrast, small‐scale studies on the dynamics of species habitat change are more conducive to species protection and determining the abiotic factors driving this change (Wang et al., 2020, 2021). A study on 405 migrating butterfly species revealed that over 85% of butterfly species show seasonal switching, 62 species will face elevated extinction risk, and the tropics exhibit more significant performance (Chowdhury et al., 2021). Thus, exploring the distribution of species at smaller time scales and determining the driving parameters are important for species conservation, especially in wild populations.

As a common traditional Chinese medicine, *Epimedium brevicornum* Maxim. (*E. brevicornum*) belongs to the genus Epimedium (Berberidaceae) and is broadly used against cardiovascular diseases, fractures, infertility, bone and joint diseases, impotence, and gonad dysfunctions (Mahboubi, 2021; Wang et al., 2003; Zhang et al., 2006; Zheng et al., 2020). *E. brevicornum* is mainly distributed in central China, such as Gansu, Shaanxi, Henan, Hebei, Shanxi, and Sichuan, covering large areas of shrubs and woodlands. The cool shade of moist coniferous forests and thickets is generally the habitat preference of *E. brevicornum*, leading to the particularity of species to the surrounding environment. For example, *E. brevicornum* is difficult to plant owing to its low seed viability, and the greatest performance will be from seeds cultivated in slightly alkaline soils that are sheltered from sunshine and at relatively high humidity (Lone et al., 2018). However, corresponding stringent light and temperature requirements for the regrowth of roots and shoots are needed during the reproductive growth period (Lone et al., 2018; Lubell & Brand, 2005). Furthermore, based on its valuable commercial market value, *E. brevicornum* is being utilized at an increasing frequency in the medical field, even more widely than *Epimedium koreanum* Nakai and *Epimedium sagittatum* (Sieb. & Zucc.) Maxim. Ultimately, the biological characteristics and high business commercial prospects of *E. brevicornum*, as well as the integrated influence of anthropogenic activities and climate changes, have resulted in the depletion of wild resources of *E. brevicornum* (Ward, 2004; Xu et al., 2008). Therefore, exploring the spatial dynamics of this valuable and promising plant over several time scales is important for the conservation of wild populations and future artificial cultivation.

As an excellent evaluation tool, species distribution models (SDMs) are widely used to simulate the potential distribution of species based on occurrence records and the characteristics of sites. For decades, the maximum entropy (MaxEnt), random forest (RF), domain, genetic algorithm for rule set production (GARP), Climex, and Domain have been highly accepted in habitat suitability assessments (Booth et al., 2014; Byeon et al., 2018; Merow et al., 2013; Townsend Peterson et al., 2007). Among them, the MaxEnt model has been widely utilized owing to its great performance with a short run time, ease of operation, and accurate simulation effects (Elith et al., 2011; Merow et al., 2013; Zhao, Cui, et al., 2021; Zhao, Deng, et al., 2021). To explore the potential distribution dynamics under climate change, Kong et al. (2021) established a climate distribution model under four climate change scenarios and revealed that isolated, fragmented giant panda populations are more vulnerable than other populations to extinction risk. Li et al. (2020) mapped the current and future distributions of three *Coptis* herbs in China and revealed that the annual precipitation range and isothermality were selected as the critical variables driving the current and future distribution patterns. To understand the adaptive strategies to climate change for *Cyclobalanopsis glauca*, Zhang et al. (2022) predicted the spatial patterns under current and future conditions by using the MaxEnt model, and the results indicated that the influence of annual precipitation will lead to an expansion of the distribution to a higher latitude. During the process of habitat suitability evaluation, the MaxEnt model can be used to determine spatial dynamic maps, driving environmental factors, and change mechanisms (Chowdhury et al., 2021; He et al., 2021; Zhang et al., 2018; Zhang, Liu, et al., 2021; Zhang, Zuo, et al., 2021). Consequently, we used this modeling method to simulate the distribution dynamics of *E. brevicornum* over five decades, and the driving parameters were identified under different conditions.

Herein, we developed the MaxEnt model over five decades to explore the spatial dynamics of *E. brevicornum* at smaller time scales, including 1970–1979, 1980–1989, 1990–1999, 2000–2009, and 2010–2019. Additionally, the Kuenm package was used to enable detailed model calibration and selection, and the ultimate parameter combination was obtained for optimal spatial distribution (Cobos et al., 2019; Yan et al., 2021). The objectives of this study were to simulate the spatial distribution dynamics over five decades for *E. brevicornum* and to determine the driving factors. These results provide a reasonable basis for clearly understanding the changes in the spatial distribution of the species over the last 50 years and provide conservation recommendations.

## 2 MATERIALS AND METHODS

### 2.1 Distribution data collection

**FIGURE 1:**
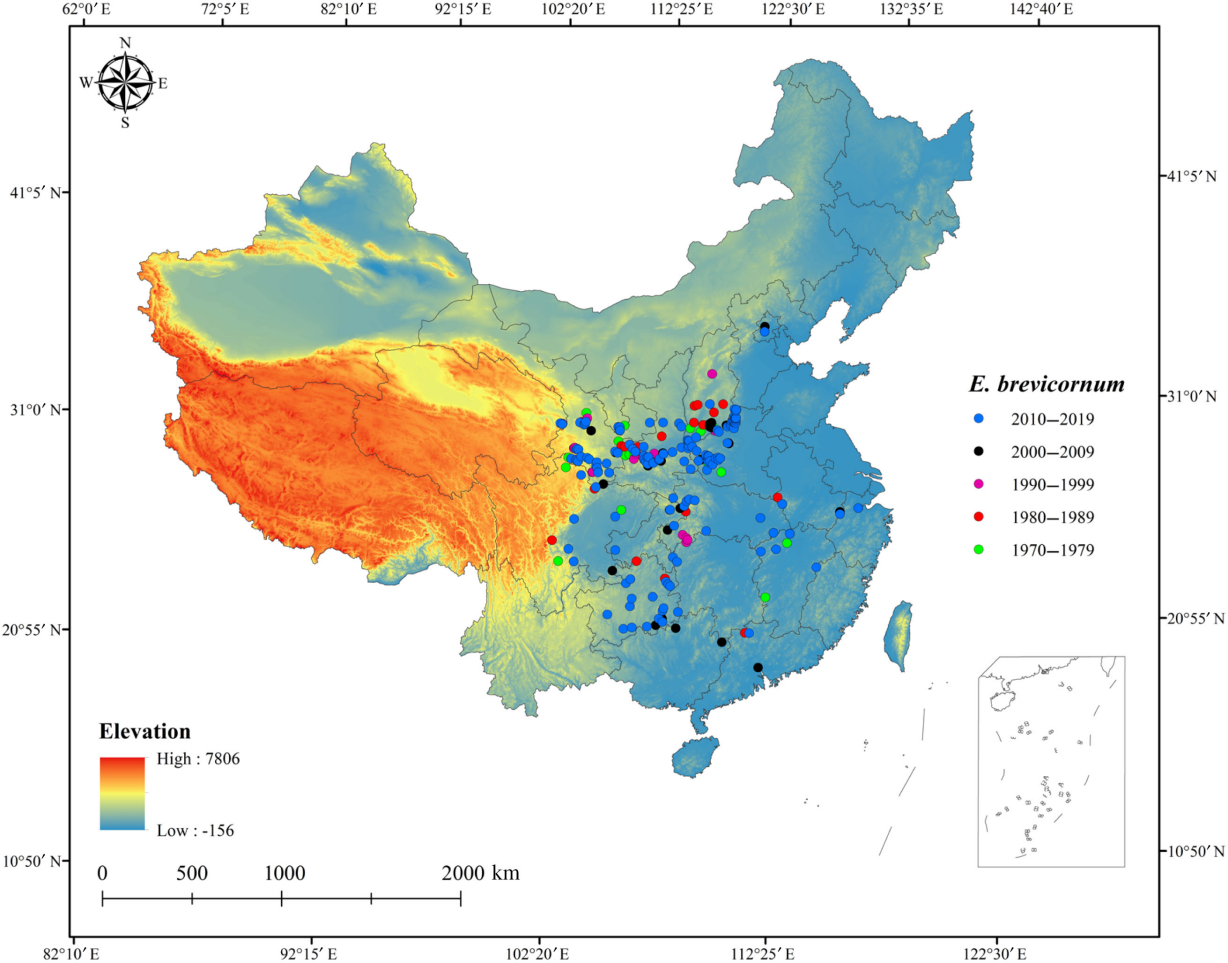
Occurrence records for *Epimedium brevicornum* for five time scales (1970–1979, 1980–1989, 1990–1999, 2000–2009, and 2010–2019).

The species distribution data were mainly collected in China by extensive literature searches, and other online databases, such as the Global Biodiversity Information Facility (https://www.gbif.org/), the Plant Photo Bank of China (http://ppbc.iplant.cn/), the Chinese Virtual Herbarium database (http://www.cvh.ac.cn/), the Specimen Resources Sharing Platform for Education (http://mnh.scu.edu.cn/), and the China National Knowledge Infrastructure (https://www.cnki.net/). During the data collection, the year of collection at the point of occurrence was used to determine the time scale. For every 10‐year scale, the distribution points without accurate location information were deleted, and Google Earth (http://ditu.google.cn/) was used to identify the distribution points with exact geocoordinate information from the records. Then, we removed duplicate distribution points, and only one point was obtained based on the resolution of environmental variables (1 km × 1 km). Consequently, 21, 30, 18, 34, and 142 distribution records of *E. brevicornum* under five time scales (1970–1979, 1980–1989, 1990–1999, 2000–2009, and 2010–2019) were obtained (Figure 1).

### 2.2 Environmental parameters

As significant driving environmental factors, 22 variables, originated from the World Climate Database 2.1 (www.worldclim.org), were collected to establish the MaxEnt model. For three monthly weather data, we obtained the maximum temperature, minimum temperature, and precipitation in five time scales (1970–1979, 1980–1989, 1990–1999, 2000–2009, and 2010–2019), respectively. All the environmental factors have been adjusted to 30‐s spatial (approximately 1 km^2^) resolution during the collection process. To avoid overfitting in the final simulation results owing to high correlation among environmental variables, we used ENMtools to filter the distribution point data and only one point in each grid cell. Next, the values of environmental variables were extracted according to the distribution data by using the ArcGIS 10.4. Finally, we utilized Pearson's correlation analysis in SPSS 22.0 to examine the correlations of 19 bioclimate variables, and only one environmental factor with |*r*| ≥ .8 was eliminated. Thus, we identified 7, 7, 7, 8, and 8 environmental parameters for five scales, 1970–1979, 1980–1989, 1990–1999, 2000–2009, and 2010–2019, respectively. Ultimately, each ten‐year scale with independent environmental parameters and all the factors were saved in “ASCII” format according to the needs of the MaxEnt model (Table 1).

**TABLE 1:** Environmental variables used in MaxEnt modeling. A, B, C, D, and E represent 1970–1979, 1980–1989, 1990–1999, 2000–2009, and 2010–2019, respectively.

Variable	Description	Unit	A	B	C	D	E	Key variables (total)
BIO1	Annual mean temperature	°C × 10	✓	✓	✓	✓	✓	✓
BIO3	Isothermally	/	**✓**	**✓**	**✓**	**✓**	**✓**	**✓**
BIO4	Temperature seasonality	/	**✓**	**✓**	**✓**	✓	**✓**	**✓**
BIO12	Annual precipitation	mm	✓	✓	✓	**✓**	✓	×
BIO15	Precipitation seasonality	/	×	×	×	✓	✓	×
TMAX	Maximum temperature	°C	✓	✓	✓	✓	✓	✓
TMIN	Minimum temperature	°C	**✓**	**✓**	✓	**✓**	**✓**	**✓**
PREC	Precipitation	mm	✓	✓	✓	✓	✓	✓

*Note*: The bold part indicates that it was selected as an important environmental factor, and the key variable is the comprehensive results of the evaluation.

### 2.3 Model calibration and MaxEnt modeling process

The results will be different under the influence of various software parameters, such as regularization multiplier (RM) and feature combinations (FCs). Abundance of studies has revealed the great influence of RM and FCs on the results of MaxEnt modeling (Bald et al., 2023; Muscarella et al., 2014; Radosavljevic & Anderson, 2014). So, we optimized the combinations of RM and FCs for five time scales by using the Kuenm package in R software (Cobos et al., 2019; Santos‐Hernández et al., 2021). Specifically, parameters with RM values between 0.5 and 4 (increments of 0.5, total of 8 values) and 31 different FCs were identified by 5 feature parameters, including linear (L), quadratic (Q), hinge (H), product (P), and threshold (T), for 248 (8 RM × 31 FC) model parameter combinations. We assessed the model parameter combination in accordance with the model complexity (AICc, the Akaike minimum information criterion), significance (partial ROC), and omission rates (*E* = 5%). Furthermore, the significant models needed to meet the following conditions: omission rates ≤5% and delta AICc values ≤2 (Cobos et al., 2019).

We performed MaxEnt modeling every 10 years using the results of parameter optimization after identifying the model parameter collocation. Under each time scale, 25% of the occurrence points were randomly selected as the test set, while 75% of the distribution data were used as the training set. We obtained the final average results after executing the MaxEnt model program with 10 replicates. Then, other parameters were set as follows: a convergence threshold of 10^−5^ was used, the maximum number of iterations was 500, a maximum number of background points equal to 10,000 was used, and “ASC” was used as the output file type. In addition, the jackknife method was used to determine the significance of important environmental factors to the *E. brevicornum* distribution for every scale, and the final output format was logistic. To evaluate the accuracy of the MaxEnt modeling, the area under the receiver operating characteristic (ROC) curve (AUC) was utilized, and the value ranged from 0 to 1. In general, AUC values greater than 0.9 indicate excellent model performance, while very good model results were obtained if the values ranged from 0.8 to 0.9. An average and poor modeling performance would be represented by AUC values of 0.7 < AUC < 0.8 and <0.7, respectively (Kaky et al., 2020; Li et al., 2022).

Ultimately, the habitat distribution maps for *E. brevicornum* for the five decade scales were visualized using ArcGIS 10.4 after obtaining the suitability of the *E. brevicornum*, and four levels of suitability, including highly suitable (0.6–1), moderately suitable (0.4–0.6), minimally suitable (0.2–0.4), and not suitable (0–0.2), were identified.

### 2.4 Changes in highly suitable areas for *E. brevicornum*

For different time scales, the variations in suitable areas, especially in highly suitable regions, indicate that the living habitat of *E. brevicornum* is different under the driving environmental conditions. The highly suitable areas represent the best habitat for the distribution and growth of *E. brevicornum*. Consequently, the ArcGIS 10.4 extraction tool was utilized to obtain the highly suitable areas, and we used SDM tools in ArcGIS 10.4 to simulate the expansion and contraction areas from 1970–1979 to 1980–1989, 1990–1999, 2000–2009, and 2010–2019 (Brown, 2014; Brown et al., 2017). Ultimately, this study mapped the change trends for four time scales, and three different types of areas, including expansion, stability, and contraction, were identified.

### 2.5 Spatial dynamics simulation

To better represent the dynamics from one period to the next, a Venn diagram was used to compare them for the highly suitable regions for *E. brevicornum*. ArcGIS 10.4 was utilized to calculate the areas and corresponding proportions of each part according to the total overlap results of the highly suitable regions.

Furthermore, the trends of changes in highly suitable areas for *E. brevicornum* were calculated and the centroids were compared for different highly suitable areas in five time scales using the ArcGIS 10.4. This study focused on obtaining a summary of the core shift in the distribution of highly suitable regions for *E. brevicornum*. Specifically, we used ArcGIS 10.4 to reduce the distribution highly suitable areas to a single centroid (central) point to depict the magnitude and direction of highly suitable areas under five time scales.

## 3 RESULTS

### 3.1 Model optimization

**FIGURE 2:**
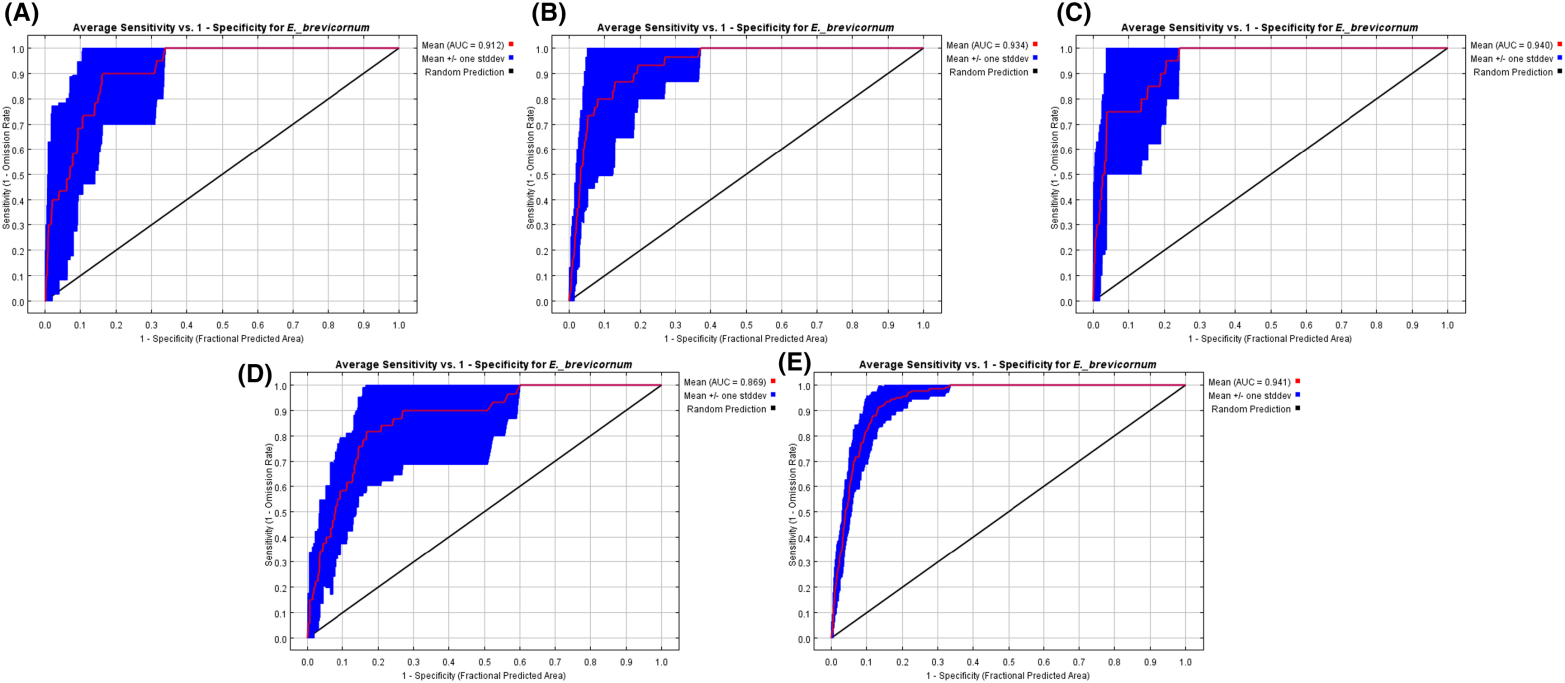
ROC curves obtained by the MaxEnt model. A, B, C, D, and E represent 1970–1979, 1980–1989, 1990–1999, 2000–2009, and 2010–2019, respectively.

Under different combinations of model parameters, MaxEnt modeling for various species populations will present significantly different performances, which will further influence the stability of the potential distribution. The default settings of FC and RM in the MaxEnt model are LQPH and 1, respectively. Therefore, the Kuenm package in R software was used in our study to optimize the parameter combinations for the five stages, and the results are presented in Table 2. Thus, we obtained different combinations for 1970–1979 (FC = Q, RM = 1.5), 1980–1989 (FC = P, RM = 4), 1990–1999 (FC = L, RM = 0.5), 2000–2009 (FC = QH, RM = 2.5), and 2010–2019 (FC = QP, RM = 0.5). Under five stages, this study executed MaxEnt modeling to identify the ultimate average results, and the mean AUC values for *E. brevicornum* were 0.912, 0.934, 0.94, 0.869, and 0.941, indicating the excellent performance of the model during this simulation (Figure 2).

**TABLE 2:** Different parameter combinations for *Epimedium brevicornum* for the five time scales. A, B, C, D, and E represent 1970–1979, 1980–1989, 1990–1999, 2000–2009, and 2010–2019, respectively.

Parameter	A	B	C	D	E
Feature combinations (FCs)	Quadratic (Q)	Product (P)	Linear (L)	Quadratic (Q), Hinge (H)	Quadratic (Q), Product (P)
Regularization multiplier (RM)	1.5	4	0.5	2.5	0.5

### 3.2 Critical environmental parameter

For *E. brevicornum*, we identified the significant environmental variables that influence the potential distribution of species under five parameter combinations, and over 80% of the total contribution rate was considered the evaluation standard. Consequently, we determined five groups of important environmental factors for *E. brevicornum* for the five stages, and the average contribution was obtained after averaging all the variables. In general, temperature seasonality (BIO4, 37.54%), minimum temperature (TMIN, 21.42%), and isothermal conditions (BIO3, 21.38%) were regarded as the most significant variables for the distribution of *E. brevicornum*, with over 80% average contributions. For 1970–1979, temperature seasonality (BIO4, 51%) was evaluated as the most critical factor under these conditions, followed by isothermal conditions (BIO3, 25.5%) and minimum temperature (TMIN, 23.4%). Then, three environmental parameters, including temperature seasonality (BIO4, 43%), minimum temperature (TMIN, 34.3%), and isothermal conditions (BIO3, 22.8%), for 1980–1989 were deemed excellent variables. For 1990–1999, temperature seasonality (BIO4, 58.1%) performed very well during the MaxEnt modeling of *E. brevicornum*, and isothermal conditions (BIO3) also seemed to be a critical factor, with a 22.4% contribution rate. During 2000–2009, the critical variables, including annual precipitation (BIO12, 47.5%), isothermal conditions (BIO3, 19.8%), and minimum temperature (TMIN, 16.8%), were assessed as the driving factors. For 2010–2019, temperature seasonality (BIO4, 35.6%), minimum temperature (TMIN, 32.6%), and isothermal conditions (BIO3, 16.4%) were considered significant parameters that influenced the distribution of this plant.

### 3.3 Spatial distribution of *E. brevicornum*

**FIGURE 3:**
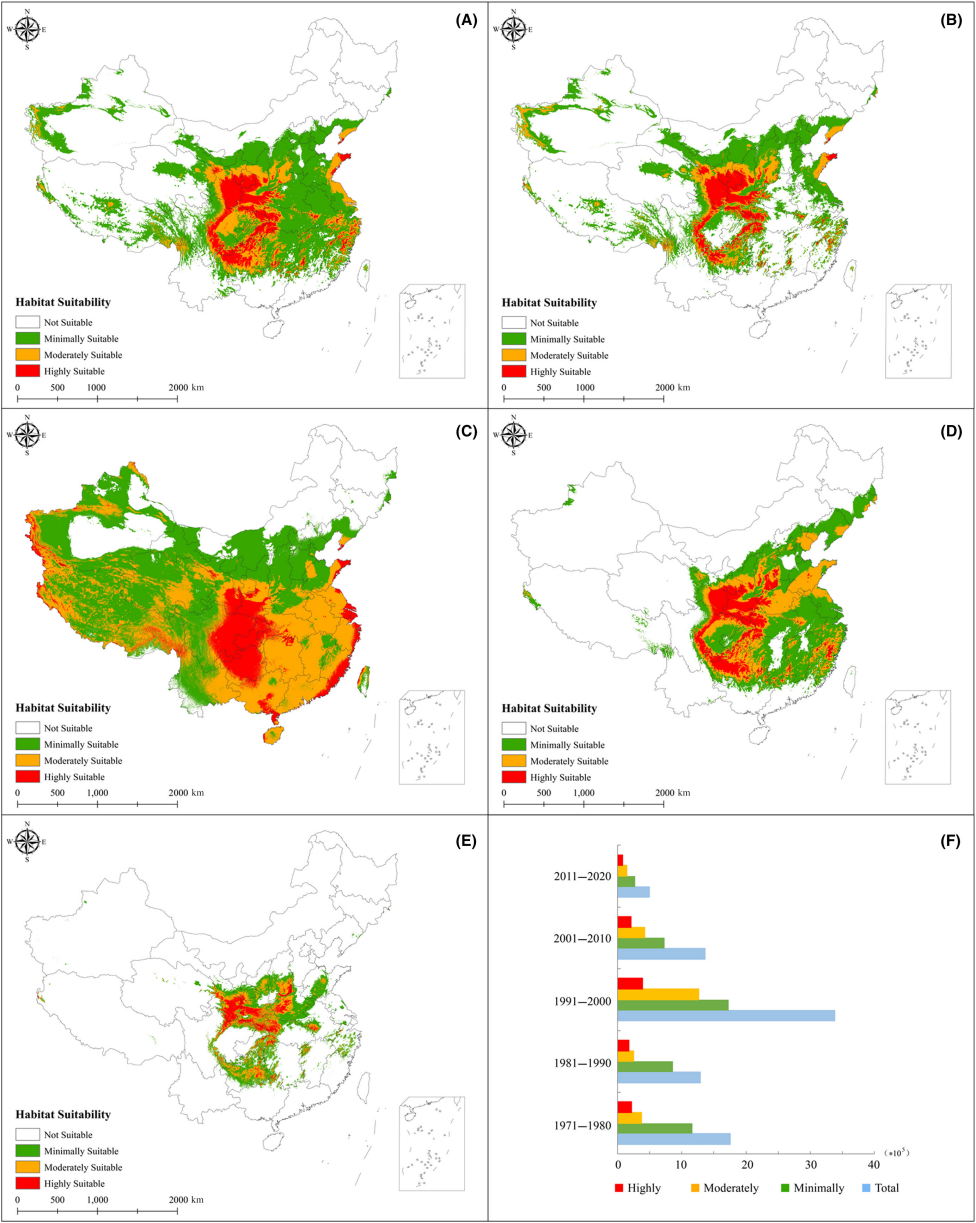
Potential distributions of *Epimedium brevicornum*. A, B, C, D, and E represent 1970–1979, 1980–1989, 1990–1999, 2000–2009, and 2010–2019, respectively. F shows the changes in area for the five scales.

Figure 3 shows the potential geographic distribution of *E. brevicornum* for the five stages, and corresponding areas were determined during the evaluation process. In general, suitable areas for *E. brevicornum* were mainly distributed in Gansu, Shaanxi, Shanxi, and Henan, with smaller distributions in Hubei, Hunan, Sichuan, Chongqing City, and Anhui. Highly suitable zones were mainly concentrated in Gansu and Shaanxi, while sporadic zones were distributed in other provinces, such as Sichuan, Chongqing City, Hubei, and Hunan. For 1990–1999, the largest area of suitability for *E. brevicornum* was identified, which was approximately 67.64 × 10^5^ km^2^, followed by 1971–1979 (approximately 35.06 × 10^5^ km^2^), 2000–2009 (approximately 27.29 × 10^5^ km^2^), 1981–1989 (approximately 25.7 × 10^5^ km^2^), and 2010–2019 (approximately 9.87× 10^5^ km^2^). The changing trends of the distribution areas of highly, moderately, and minimally suitable regions were similar to those of the suitable regions, and in general, they decreased first, then increased, and finally decreased. For 1970–2019, the high suitability zones underwent five major changes, with areas from 4.35 × 10^5^ km^2^ to 3.5 × 10^5^ km^2^, 7.79 × 10^5^ km^2^, 4.22 × 10^5^ km^2^, and 1.55 × 10^5^ km^2^, which colonized corresponding suitable areas of 4.73%, 3.81%, 8.48%, 4.59%, and 1.69%, respectively. For moderate regions for 1970–1979, 1980–1989, 1990–1999, 2000–2009, and 2010–2019, areas of 7.48 × 10^5^ km^2^, 5.02 × 10^5^ km^2^, 25.24 × 10^5^ km^2^, 8.37 × 10^5^ km^2^, and 2.91 × 10^5^ km^2^ were obtained, amounting to 8.14%, 5.46%, 27.47%, 9.11%, and 3.17%, respectively.

### 3.4 Expansion and contraction of highly suitable zones

**FIGURE 4:**
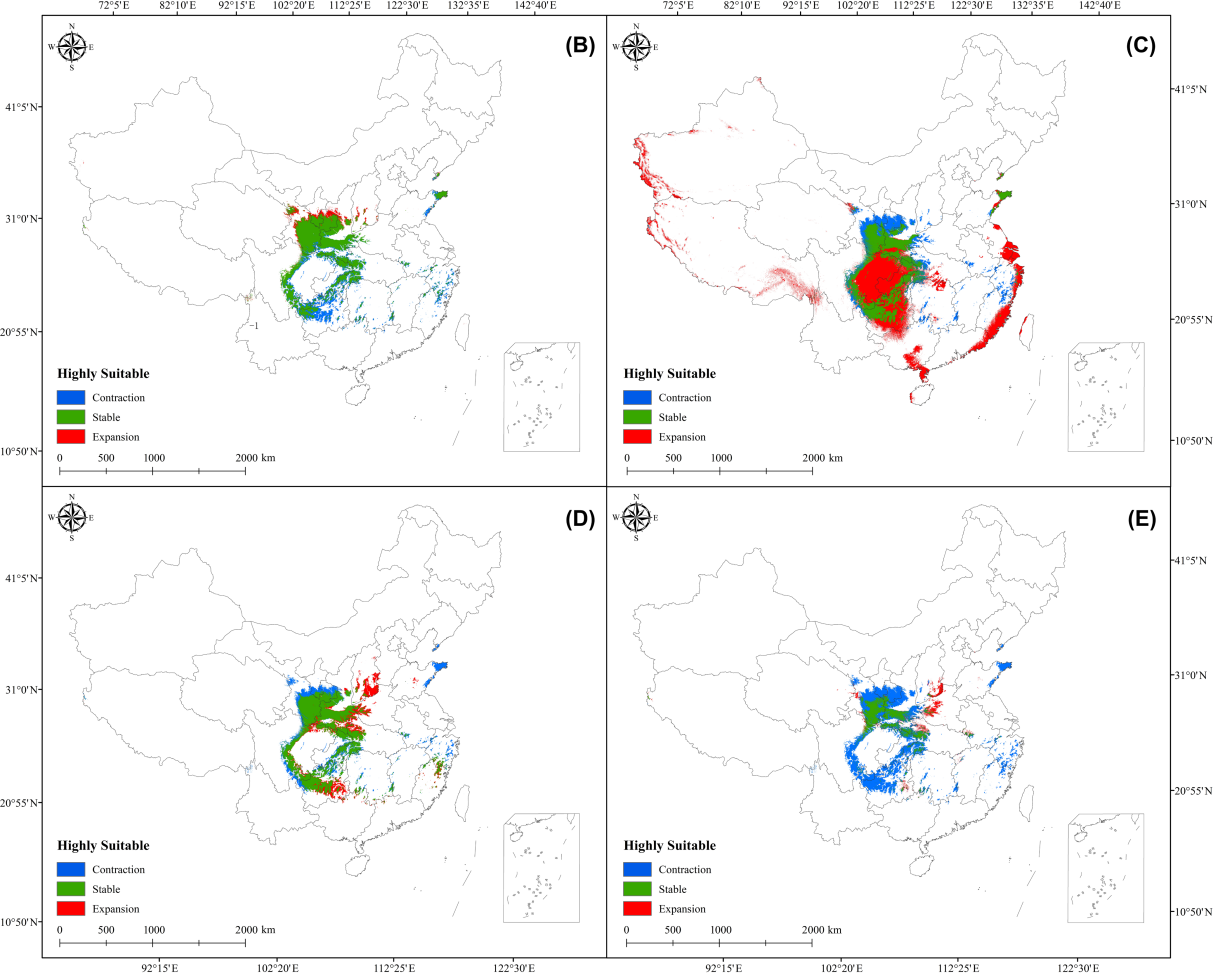
Areas of expansion, stability, and contraction for the four scales. B, C, D, and E represent 1980–1989, 1990–1999, 2000–2009, and 2010–2019, respectively.

After evaluating the characteristics of suitable distributions for *E. brevicornum*, the highly suitable habitats for the five stages were identified to compare the changes, and Figure 4 shows this result. Overall, the stable regions were mainly concentrated in southern Gansu and western and southern Shaanxi, with sporadic distributions in central Sichuan and part of Chongqing city. For the expansion areas of *E. brevicornum*, southeastern Gansu and central Sichuan were the most obvious, and large new suitable regions were observed in these areas. Additionally, the contraction areas were mainly concentrated at the junction of Sichuan and Guizhou, as well as at the junction of Shaanxi, Hubei, and Henan. From 1970–1979 to 1980–1989, a total of 0.35 × 10^5^ km^2^, 3.4 × 10^5^ km^2^, and 1.32 × 10^5^ km^2^ that were increased, stable, and decreased, respectively, were obtained. For 1990–1999, we determined 5.6 × 10^5^ km^2^ of new expansion regions, and approximately 3.01 × 10^5^ km^2^ of areas remained stable. From 1971–1980 to 2000–2009, the areas of expansion, stability, and contraction for *E. brevicornum* were 1.06 × 10^5^ km^2^, 3.54 × 10^5^ km^2^, and 1.19 × 10^5^ km^2^, respectively. Then, we identified regions of approximately 0.41 × 10^5^ km^2^ and 3.47 × 10^5^ km^2^ that increased and decreased during 2010–2019, respectively, while the stable area was approximately 1.25 × 10^5^ km^2^. For these results, the area that changed the most in terms of expansion and stability occurred in 1990–1999, and the most contracted regions were in 2010–2019.

### 3.5 Spatial dynamics of highly suitable zones

**FIGURE 5:**
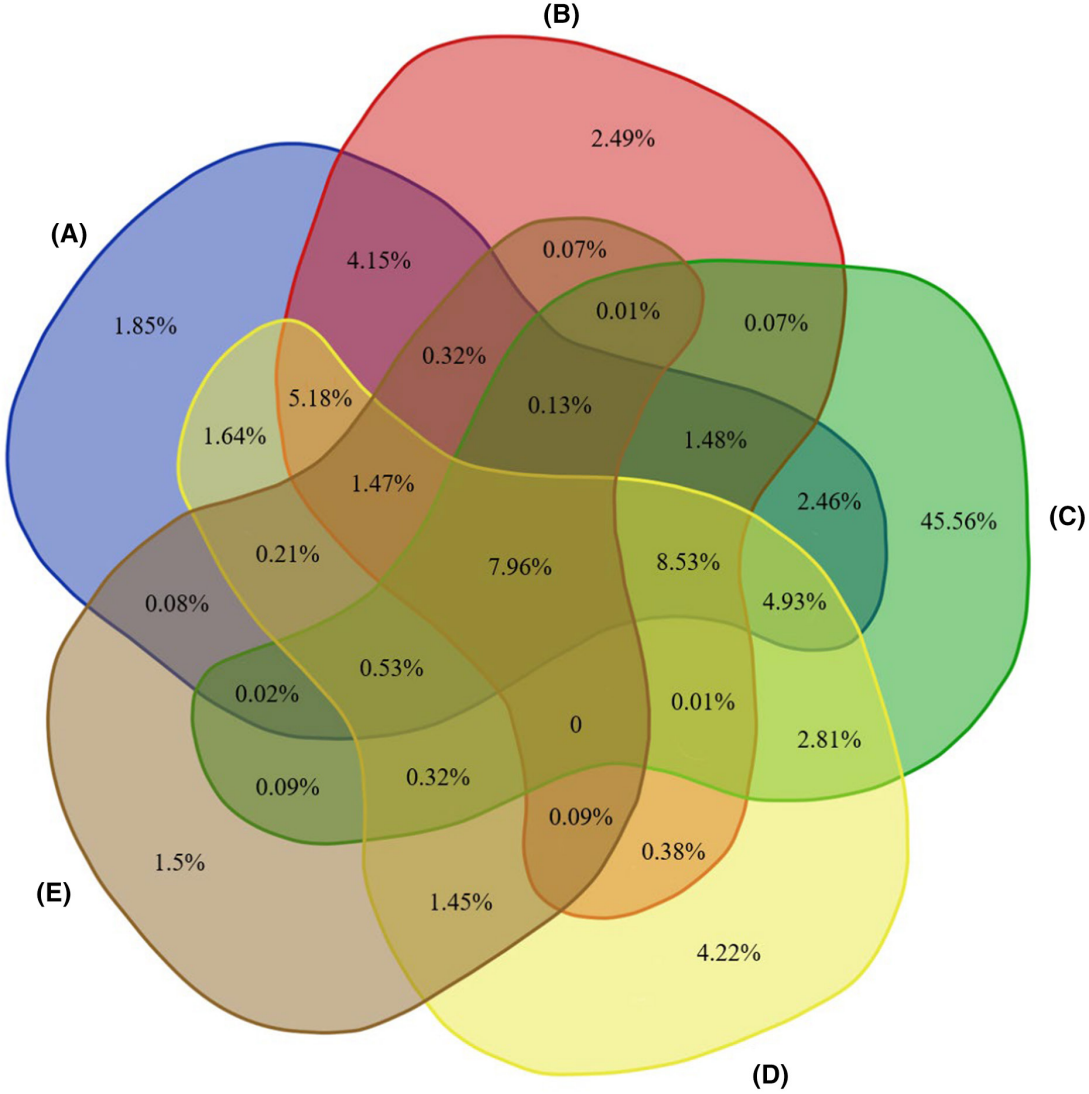
Spatial distribution proportions of *Epimedium brevicornum* for the five scales. A, B, C, D, and E represent 1970–1979, 1980–1989, 1990–1999, 2000–2009, and 2010–2019, respectively.

The Venn diagram indicates that approximately 7.96% of the highly suitable areas are very beneficial to the distribution of *E. brevicornum* during the five stages (Figure 5). From A to B, 3.12% of the highly suitable areas increased, while approximately 11.72% of the regions disappeared for different reasons. Among them, 2.49% and 1.85% of the highly suitable habitat belonged to completely new expansion and contraction regions, respectively. Then, 13% of net increased areas for *E. brevicornum* was obtained when transitioning to the third stage. In addition, approximately 45.56% of highly suitable regions for the five stages were retrieved, indicating excellent performance for the distribution of *E. brevicornum* in 1990–1999, followed by 2000–2009, 1980–1989, 1970–1979, and 2010–2019.

### 3.6 The core distributional shifts

**FIGURE 6:**
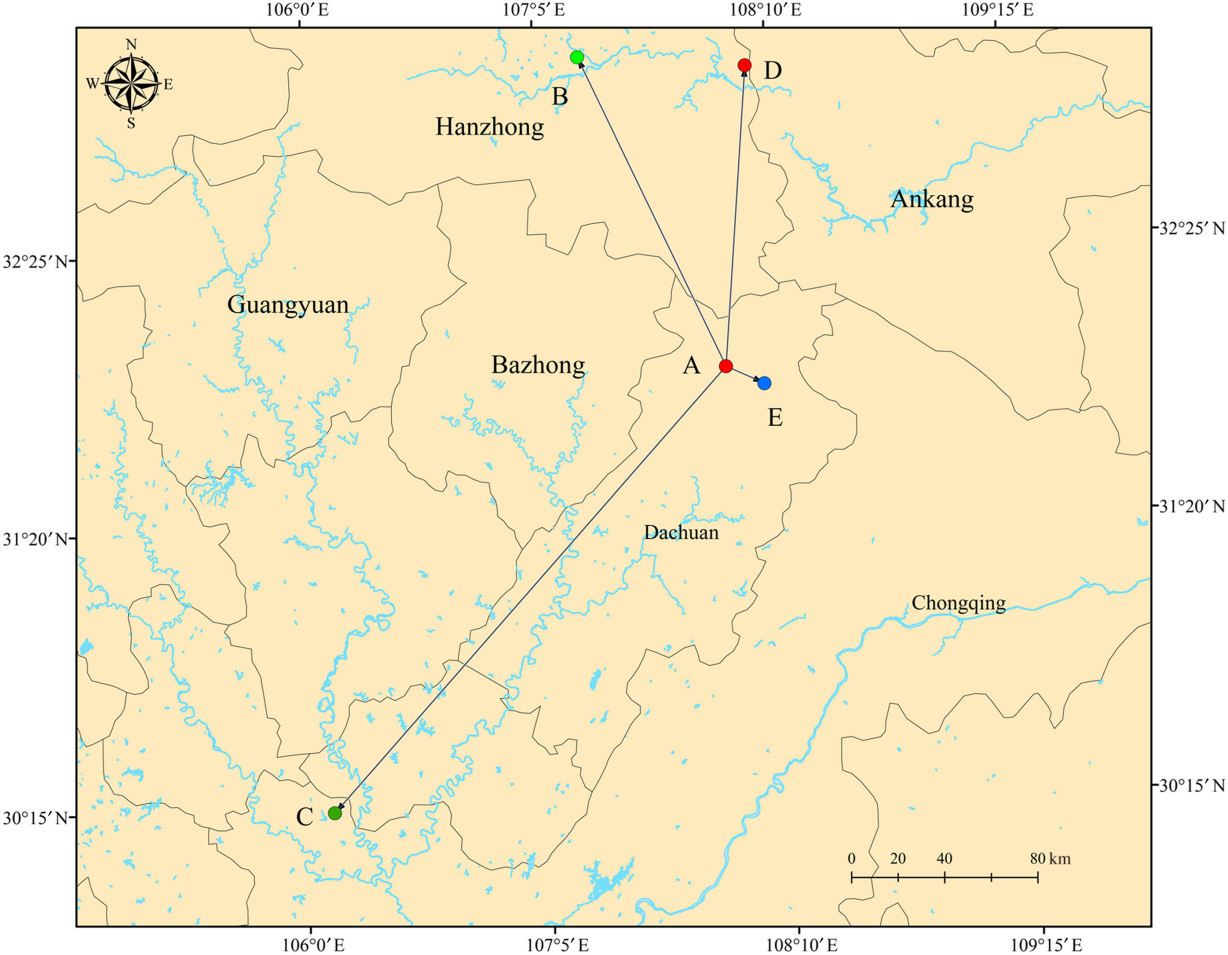
The changing trends of the spatial distributions for the five time scales. A, B, C, D, and E represent 1970–1979, 1980–1989, 1990–1999, 2000–2009, and 2010–2019, respectively.

In this study, the centroid of the highly suitable habitat was used to indicate the comprehensive geographic position of *E. brevicornum* from 1970 to 2020. The centroid of the highly distribution areas was located in Wanyuan County in Dachuan City in Sichuan Province during 1970–1979 (107.93 E, 31.96 N). Then, the core of highly suitable regions was predicted to shift to 107.29 E, 33.19 N in Chenggu County in Hanzhong City (migration distance of 146.2 km) during 1980–1989. For 1990–1999, the centers of *E. brevicornum* shifted to Hechuan County in Chongqing City (106.11 E, 30.26 N; migration distance of 256.04 km). For 2000–2009 and 2010–2019, the simulated cores of the highly suitable habitat for *E. brevicornum* were located in Xixiang County in Hanzhong City in Shaanxi Province (108.11 E, 31.89 N) and Wanyuan County in Dachuan City in Sichuan Province (108.07 E, 33.13 N), respectively, while their shift distances were 184.18 km and 127.41 km, respectively (Figure 6). Interestingly, the shift direction of *E. brevicornum* under different environmental stressors is mostly located near rivers, indicating that the role of precipitation in the development and distribution of *E. brevicornum* also cannot be ignored.

## 4 DISCUSSION

### 4.1 Model performance after optimization

In this study, we developed a time niche model for *E. brevicornum*, and the Kuenm package in R software was used to optimize the MaxEnt parameter combinations for five different time periods (1970–1979, 1980–1989, 1990–1999, 2000–2009, and 2010–2019). Then, we simulated the spatial distribution of *E. brevicornum* and determined the corresponding environmental factors by constructing different parameter combinations. Our results improved the accuracy of MaxEnt modeling for *E. brevicornum* compared with previous studies (Li et al., 2021, 2023; Ma et al., 2020), and more credible results were obtained. Firstly, we constructed the MaxEnt model in detail under different time scales using the more accurate data. Compared with previous distribution modeling studies of *E. brevicornum* in which only occurrence data, environmental variables, and default parameter settings were used (Li et al., 2021, 2023; Ma et al., 2020), the model performance was optimized by using the Kuenm package, and different time scales were utilized to evaluate the spatial dynamics of the species. A number of studies have revealed that the default model parameters will bring instability to simulation results, and the model performance will be improved after adjusting the parameter combinations (Cobos et al., 2019; Obiakara et al., 2020; Zhao, Cui, et al., 2021; Zhao, Deng, et al., 2021). The response curves are significantly smoother and the AUC values are higher than those in previous studies, indicating that the distribution of species will be better if an adjusted model is used during the simulation, which is consistent with other studies (Bao et al., 2022; Santos‐Hernández et al., 2021).

### 4.2 Significant environmental variables

For *E. brevicornum*, this study identified the important factors over five time periods based on different occurrence records, revealing that the species distribution will further influence the modeling results. The temperature seasonality, minimum temperature, and isothermal conditions are the most significant variables driving the distribution patterns of *E. brevicornum*. The local habitat suitability of plants is influenced by environmental variables, such as temperature and precipitation. As the most important factors, temperature, especially temperature seasonality (BIO4), made substantial contributions to the modeling of *E. brevicornum*. A number of studies indicate that temperature greatly affects the distribution of plants, and the physiological characteristics of plants constantly change in response to changes in ambient temperature (Franklin, 2009; Moles et al., 2014; Randin et al., 2009). Under different dimensional gradients, the distribution and response of plants to the local environment are mainly limited by temperature, and the role of temperature is much greater than that of precipitation (Bertin, 2008; Woodward, 1988). Studies have revealed that some phenological events, including leafing and flowering, will typically advance if they become warmer earlier, while these events will be the opposite in autumn (Bertin, 2008; Forrest, 2016; Singh et al., 2017). In addition, temperature signals are transmitted to cells to stimulate them to respond to changes in external temperature under constantly changing external temperature (Ruelland & Zachowski, 2010), which will prompt plants to readjust their biochemical composition to adapt to the changing environment. For *Epimedium* species, temperature also plays a key role in seed germination, plant traits, and the accumulation of secondary metabolites (Liu, 2020; Su, 2016; Zhang & Fan, 2016). For example, the proper temperature will break the dormancy of *E. sagittatum* seeds when transferred from higher temperatures, and the germination rate will increase (Du, 2017; Wang, 2022). In Sichuan Province, the time and amount of fruit ripening for *E. brevicornum* are closely related to environmental factors under different altitudes, and abrupt changes in temperature may account for the difference in the first place (Wu et al., 2008). The reasons why the performance of BIO4 is better than those of BIO1 and BIO3 may originate from the fact that the seasonal effect of temperature on *E. brevicornum* is greater than average.

### 4.3 Distribution and spatial dynamics over the five time scales

For thousands of years, the main production area of *E. brevicornum* has been changing, indicating that it has great adaptability and is substantially affected by local habitat conditions. Therefore, the prediction of spatial dynamics for *E. brevicornum* is highly useful for current and future artificial planting and management. Over the five time scales, the suitable distribution of *E. brevicornum* represented a difference and was different at various levels of suitability. Southern Gansu, central Sichuan, Guizhou, Hubei, and Henan have become the core regions of change, and shrinking areas are the focus of conservation. For 1990–1999, approximately 5.6 × 10^5^ km^2^ and 1.71 × 10^5^ km^2^ of expansion and contraction zones were obtained, respectively, which may have resulted from substantial artificial disturbance or global climate changes. After the implementation of a series of nature conservation policies, the diversity of species is significantly higher during this period, which will significantly improve the habitat quality of *E. brevicornum*. In 2010–2019, our study identified about 3.47 × 10^5^ km^2^ contraction regions owing to the climate change. In addition, a Venn diagram becomes a useful tool to show the changing proportion of highly suitable regions over different time scales, and every patch represents a rate of area change. Over the five scales, each patch was the overlapping result of the high distribution areas for *E. brevicornum*, and the patch was more stable if it overlapped for a longer period of time. Thus, this study ultimately determined approximately 7.96% of regions, which is the result of five overlays. Most of these regions may belong to table change zones at the five time scales. Additionally, Hanzhong City in Sichuan Province may represent the best region under current conditions, and the accurate location is 108.07 E, 33.13 N, which was indicated by the results of core distributional shifts. Notably, Shaanxi Province was the core of highly suitable regions for 1980–1989 and 2000–2009, while Sichuan Province became a significant region for the distribution of *E. brevicornum* in other time scales, indicating that corresponding conservation and management measures should be considered. Studies have shown that the climate of the Sichuan Basin is suitable for the survival of most species, and the biodiversity is very high (Lu et al., 2012; Yang et al., 2022), which may be a critical reason for the continuous movement of the suitable area to the southwest.

### 4.4 Conservation and planning recommendations

To better protect the wild *E. brevicornum* population and benefit artificial cultivation, this study proposes the following strategies. According to the results of these studies, managers must implement different planning recommendations based on potential distribution for stability, expansion, and contraction. Apparently, all three categories of areas need to be protected, especially contraction, followed by stable areas. Stable areas, such as southern Gansu, western and southern Shaanxi, and part of Chongqing City, should be prioritized for introduction and cultivation under slight human interference, which is conducive to habitat conservation for *E. brevicornum*. In these regions, it is important to ensure the scale and yield of *E. brevicornum* cultivation, while wild populations should be further protected. Therefore, overplanting *E. brevicornum* in these regions is unsuitable, and researchers should work with local governments to rehabilitate the habitat. Expansion areas, including southern Gansu and central Sichuan provinces, represent the dynamic direction at each time scale, and new planting sites can be considered. Additionally, more attention should be given to some important areas, such as the junction of Sichuan and Guizhou and the junction of Shaanxi, Hubei, and Henan, which are contraction regions. Cross‐border protection has great difficulties, and local governments should strengthen communication and cooperation; these decreased areas can be used as demonstration zones for the protection of *E. brevicornum*. Awareness of *E. brevicornum* can be spread among local farmers to guide them to protect *E. brevicornum* and not overharvest it, especially wild populations.

During the process of conservation and planning management, the flexible use of critical environmental variables over the five time scales has been correspondingly important. The variables related to temperature, such as the temperature seasonality, annual mean temperature, isothermal conditions, and minimum temperature, should be considered during the whole process. Especially for temperature seasonality, managers need to keep an eye on the state of *E. brevicornum* when the seasons change. Two seasons, including spring and autumn, are very important for the growth of *E. brevicornum* because the returning green stage will occur in March, and August and September are the best harvest periods for *E. brevicornum* apples (Chen et al., 1996; Qiu et al., 2015). The accumulation of SMs in the harvest period determines the quality and yield of *E. brevicornum*, and it is especially important to ensure suitable and sufficient temperatures during these stages. Of course, the ground frost frequency, altitude, solar radiation, wind, precipitation, and land cover cannot be ignored during conservation and planting. In accordance with the role of the temperature, we should pay substantial attention to it in the production practice of *E. brevicornum* to provide seeds with enough activity. In addition, more measures, such as fertilization, sprinkling, and greenhouses, should be used to help the growth of *E. brevicornum*, and human interference with the habitat of *E. brevicornum* must be strictly controlled, especially in expansion and contraction areas. Notably, this study identified three counties, Wanyuan, Chenggu, Hechuan, and Xixiang, and protection strategies will play a potentially critical role in these places since the migration targets and directions are all directed at these sites. In the process of conservation and planning, managers must pay attention to the changes in distribution and the impact of various environmental factors on the species to ensure the sustainable use of resources.

## 5 CONCLUSIONS

For the different time scales studied, the different distribution models of *E. brevicornum* combined with distinct environmental variables were successfully developed, and corresponding conservation and planning recommendations were proposed to facilitate management. Our study indicated that different variables will play different roles over different time periods and that the chosen occurrence points will further influence the distribution modeling. This study determined critical factors, such as BIO4, TMIN, and BIO3, which play different roles during different time scales. Then, the stability, expansion, and contraction of highly suitable areas were obtained by using SDM tools. Ultimately, we proposed many measures regarding the protection and cultivation of *E. brevicornum* based on the results of distribution and critical environmental evaluation, which are conducive to improving species ecological values. Our work will provide useful management and planning advice to decision‐makers, which will facilitate the study of medicinal plants by other researchers. Of course, more detailed sampling efforts and better matching of environmental factors would lead them to obtain more accurate results during the modeling.

## AUTHOR CONTRIBUTIONS

**Yunfeng Li:** Conceptualization (equal); data curation (equal); resources (equal); software (equal). **Yan Wang:** Investigation (equal); methodology (equal); supervision (equal); visualization (equal). **Xiaojuan Du:** Formal analysis (equal); funding acquisition (equal); investigation (equal); supervision (equal). **Chunying Zhao:** Supervision (equal); validation (equal); writing – original draft (equal). **Ping He:** Conceptualization (equal); investigation (equal); supervision (equal); validation (equal). **Fanyun Meng:** Project administration (equal); visualization (equal); writing – original draft (equal); writing – review and editing (equal).

## CONFLICT OF INTEREST STATEMENT

The authors declare that they have no competing interests.

## Data Availability

Climate data and MaxEnt input files: https://datadryad.org/stash/share/0E1DiyM4zm2QjCFULBYtg0WLJ9fwXz‐40WOmY1Nmytg.
